# The Work of the Holy Ghost

**Published:** 1887-06-25

**Authors:** 


### Words of Consolation.

[Communications for this column should be addressed, "Chaplain," care of the Editor of The Hospital, who will gratefully welcome any suggestions or inquiries from matrons, nurses, and the staff generally.]

### XXXVIII.—The Work of the Holy Ghost.

There is another form of the Holy Spirit's guidance to which we now pass. He guides through conscience. In this way He will guide us into all truth, in so far as we need counsel, and a right judgment in the manifold difficulties which beset our path to heaven when once we have chosen to walk along it. But let us be quite clear what this guidance through conscience means. It is awfully misunderstood. Everybody has got a conscience of some kind: that is, a voice within, speaking with more or less distinctness, and which is either listened to or disregarded. But not everyone, by any means, has a conscience under the direction of the Holy Ghost. Conscience may be far from dead to the Spirit, yet equally far from being under His direction.

Now there are numbers of people who fancy (and a grievous error it is) that because they have got a conscience, everything or anything which that conscience allows them to do must be right. "My conscience tells me I may do this, or does not forbid me, therefore it is allowable; or my conscience tells me I need not do that, so I may be excused." Ah! this would be all very true if conscience had never been perverted or darkened in any way, if God had always reigned there, and retained His supremacy; or it may be true even now, if the whole life of the disciple is so far consecrated and sanctified as to admit of the Holy Spirit's supremacy, but not otherwise. Conscience with some people is, after all, only another name for self-will. If conscience (so called) must always be right, how is it that you will find, say, two persons under the same circumstances, and in similar positions, with consciences apparently permitting them to do totally different things, as different as light from darkness? The truth is this. When our first parents fell into sin and forfeited a greater part of their likeness to God, the whole human nature contained in them, conscience included, partook of the consequences of the Fall. When we speak of being "born in sin," we mean that we are born with something wrong about us in body, soul, and spirit. Are we to suppose then that, starting with a nature so much injured, somehow or other just that part of us we call conscience was not injured too? Are we to suppose that other portions of our moral faculty can be disorderd, and yet conscience left entirely right? No. Conscience was not totally blinded by the Fall, but it was sadly deceived and darkened, so it ceased to be the infallible guide it was probably once intended to be. When, therefore, it became so liable to error, with the rest of our nature, of course it needed some one to renew it, enlighten it afresh, and then to guide it into truth again. So when we hear people talking about their being so surely right in acting according to the dictates of conscience, we reply, "That depends entirely upon how far your conscience has been obediently given up to the Spirit's guidance, and upon how often His counsel is sought in earnest prayer."

(*To be continued.*)

